# Tracheal microbiome and metabolome profiling in iatrogenic subglottic tracheal stenosis

**DOI:** 10.1186/s12890-023-02654-7

**Published:** 2023-09-26

**Authors:** Zeqin Fan, Lihui Zhang, Li Wei, Xiaoxian Huang, Mei Yang, Xiqian Xing

**Affiliations:** Department of Respiratory and Critical Care Medicine, Affiliated Hospital of Yunnan University, Kunming, China

**Keywords:** Subglottic tracheal stenosis, Tracheal microbiome, Metabolome

## Abstract

**Background:**

To study the role of microecology and metabolism in iatrogenic tracheal injury and cicatricial stenosis, we investigated the tracheal microbiome and metabolome in patients with tracheal stenosis after endotracheal intubation.

**Methods:**

We collected 16 protected specimen brush (PSB) and 8 broncho-alveolar lavage (BAL) samples from 8 iatrogenic subglottic tracheal stenosis patients, including 8 PSB samples from tracheal scar sites, 8 PSB samples from scar-free sites and 8 BAL samples, by lavaging the subsegmental bronchi of the right-middle lobe. Metagenomic sequencing was performed to characterize the microbiome profiling of 16 PSB and 8 BAL samples. Untargeted metabolomics was performed in 6 PSB samples (3 from tracheal scar PSB and 3 from tracheal scar-free PSB) using high-performance liquid chromatography‒mass spectrometry (LC‒MS).

**Results:**

At the species level, the top four bacterial species were Neisseria subflava, Streptococcus oralis, Capnocytophaga gingivals, and Haemophilus aegyptius. The alpha and beta diversity among tracheal scar PSB, scar-free PSB and BAL samples were compared, and no significant differences were found. Untargeted metabolomics was performed in 6 PSB samples using LC‒MS, and only one statistically significant metabolite, carnitine, was identified. Pathway enrichment analysis of carnitine revealed significant enrichment in fatty acid oxidation.

**Conclusion:**

Our study found that carnitine levels in tracheal scar tissue were significantly lower than those in scar-free tissue, which might be a new target for the prevention and treatment of iatrogenic tracheal stenosis in the future.

## Introduction

Subglottic tracheal stenosis (SGS) is characterized by fibroinflammatory stenosis of the upper airway, which results in varying degrees of dyspnea. Iatrogenic subglottic tracheal stenosis (ITS), as a result of endotracheal intubation or tracheotomy, is the most common type of SGS. Other etiologies include idiopathic or autoimmune diseases or local radiation [1]. With the development of medical technology and the progress of critical care technology in China, the incidence of ITS has increased gradually [2]. Although endoscopic intervention has developed rapidly in recent years and has become the main treatment for various types of airway stenosis, frequent recurrence of tracheal stenosis requires repeated intervention, leading to significant psychological, physical and financial stress for patients [3, 4]. Therefore, the exploration of the pathophysiological mechanism of ITS may provide a potential treatment.

The pathogenesis of ITS is complex, involving aberrant inflammatory immune activation and an abnormal healing response [5]. Endotracheal intubation may cause mucosal damage, ischemia and hypoxia, which may further lead to changes in the local microenvironment and epithelial cell metabolism pattern, and the inflammatory response may be activated [5, 6]. Recent studies have emphasized the relationship between inflammation and imbalance of airway microbiota [7–9]. For example, a drug-eluting endotracheal tube was shown to prevent bacterial inflammation in subglottic stenosis by modulating the local microbiota [10]. In addition, the metabolism of narrow tissue also changed. Previous studies showed that metabolic inhibition by glutaminase inhibitors reduced cell proliferation by inhibiting glycolysis in scar fibroblasts [11]. Moreover, a histological study revealed that submucosal fibroblasts and collagen fibers proliferated in the stenotic lesion, which led to granulation tissue and scar formation [12]. Moreover, growing evidence has confirmed the infiltration of lymphocytes, macrophages, interleukin-17 A, interleukin-23, tumor necrosis factor, and transforming growth factor in stenotic tissues [13–15]. In recent years, an increasing number of studies have revealed the role of disordered microbes in inflammatory diseases, especially in the intestinal microbiome and metabolome [16–18]. However, only a few studies have explored the airway microbiome in patients with tracheal stenosis [7, 10, 19], and the role of the tracheal microbiome and metabolome in tracheal stenosis is unclear.

As part of the “omics” technologies, metabolomics and genomics greatly expand our understanding of the relationship between intestinal microorganisms and diseases by combining the two “omics” technologies. However, the role of the airway microbiome and metabolome in airway stenosis remains unclear. Therefore, we combined metagenomic next-generation sequencing (mNGS) and high-performance liquid chromatography‒mass spectrometry (LC‒MS) metabolomic technologies to evaluate the tracheal microbiome and metabolome in patients with tracheal stenosis after endotracheal intubation.

## Methods

### Patient selection

Eight iatrogenic subglottic tracheal stenosis patients who presented to the Department of Respiratory and Critical Care Medicine, Affiliated Hospital of Yunnan University, were recruited. The degree of airway stenosis was graded according to the classification of benign central airway stenosis published in the European Journal of Respiratory Medicine by Freitag et al., 2007 [20]. The inclusion criteria included a history of endotracheal intubation, grade III-IV tracheal stenosis, and no history of antibiotic use or respiratory tract infection in the past 1 month. The exclusion criteria were as follows: under 18 years of age; pregnant women; and noniatrogenic airway stenosis.

### Sample collection

All bronchoscopies were performed by the same experienced bronchoscopist. Before bronchoscopy, patients were given midazolam (0.05 mg/kg) and sufentanil citrate (1ug/kg) for moderate sedation and analgesia. Local anesthesia with 2% lidocaine was applied sequentially during the procedures. Then, a flexible bronchoscope (Olympus, BF-260) entered the trachea through one nostril. PSB samples were collected from tracheal scar-free and scar sites with a sheath brush (Olympus BC-5 CE, Olympus Imaging, Center Valley, PA, USA) through a flexible bronchoscope. The sampling positions of the scar-free PSB were located 2 cm below the scar sites. Then, the brush was pushed out of the sheath, cut with ethanol-disinfected scissors, and placed in an Eppendorf tube containing 1.5 ml of saline solution. Simultaneously, PSB sample collection was performed. BAL samples were obtained by lavaging the subsegmental bronchi of the right-middle lobe with approximately 50 ml of 0.9% NaCl solution through the bronchoscope. The first 20 ml was discharged to avoid contamination, whereas the remaining samples were collected for detection.

### Metagenomic next-generation sequencing

#### (1) Library Preparation and sequencing

For PSB and BAL samples, DNA or RNA sequencing was performed. DNA or RNA sequencing PCR-free library preparation was prepared by reverse transcription (for RNA), enzymatic fragmentation (except for plasma), end repair, terminal adenylation, and adaptor ligation. The concentration of libraries was quantified by real-time PCR (KAPA) and pooled. Shotgun sequencing was performed on the Illumina Nextseq platform. Approximately 20 million 75 bp single-end reads were produced for each library. For each run, one negative control (artificial plasma mixed with fragmented human genomic DNA) and one positive control (a mixture of inactivated bacteria, fungi, and pseudoviral particles containing synthesized DNA or RNA fragments of adenovirus and influenza A virus, respectively) were included for quality control [21, 22].

#### (2) Bioinformatic pipeline

Raw sequencing data were analyzed by a bioinformatic pipeline, which included the following steps: (1) unnecessary adapter sequences and low-quality bases (Q-score cutoff, 20) were trimmed in the pipeline; (2) human host sequences were eliminated by mapping to the human reference genome (GRCh38.p13 https://www.ncbi.nlm.nih.gov/assembly/2334371) using BWA (BurrowsWheeler alignment); and (3) after removal of low-complexity reads, the remaining sequencing data were simultaneously aligned by BWA to a reference database [NCBI nt database and GenBank (Benson et al., 2013)] to identify microbial species [23].

#### (3) mNGS reporting criteria

Microbial reads identified from a library were reported if (1) the sequencing data passed quality control filters (library concentration > 50 pM, Q20 > 85%, and Q30 > 80%); (2) negative control (NC) in the same sequencing run does not contain the species or the RPM (sample)/RPM (NC) ≥ 5, which was determined empirically as a cutoff for discriminating true positives from background contaminations [24].

#### (4) Analysis of mNGS data

The differential abundance of bacterial taxa between groups was assessed using the Wald test within DESeq2 v1.20.030 based on read counts scaled to account for genome size with the Benjamini–Hochberg adjustment for multiple comparisons. Relative abundance was calculated using scaled read counts as a fraction of total nonhost reads per sample. Alpha diversity was calculated using QIIME v1.8.090 with counts normalized using the size-factor method implemented within the R package DESeq2 v1.22.230. A.

### LC‒MS metabolomics

#### (1) Sample preparation and extraction

The sample stored at -80 °C was thawed on ice and vortexed for 10 s. A 150 µL extract solution (ACN:methanol = 1:4, V/V) containing internal standard was added to a 50 µL sample. Then, the sample was vortexed for 3 min and centrifuged at 12,000 rpm for 10 min (4 °C). A 150 µL aliquot of the supernatant was collected and placed at -20 °C for 30 min and then centrifuged at 12,000 rpm for 3 min (4 °C). Then, 120 µL aliquots of supernatant were transferred for LC‒MS analysis.

#### (2) HPLC conditions (T3)

All samples were acquired by the LC‒MS system following the manufacturer’s instructions. The analytical conditions were as follows: UPLC: column, Waters ACQUITY UPLC HSS T3 C18 (1.8 μm, 2.1 mm*100 mm); column temperature, 40 °C; flow rate, 0.4 mL/min; injection volume, 2 µL; solvent system, water (0.1% formic acid):acetonitrile (0.1% formic acid); gradient program, 95:5 V/V at 0 min, 10:90 V/V at 11.0 min, 10:90 V/V at 12.0 min, 95:5 V/V at 12.1 min, 95:5 V/V at 14.0 min.

#### (5) Metabolomic Data Analysis

The orthogonal partial least squares analysis (OPLS-DA) model was used to distinguish the metabolic profiling of two groups of PSB samples. The heatmap was generated by r (v 3.6.1). HMDB pathway enrichment analysis of the differentially abundant metabolites was performed using R (v 3.6.1).

## Results

### mNGS results of all samples

**Fig. 1:**
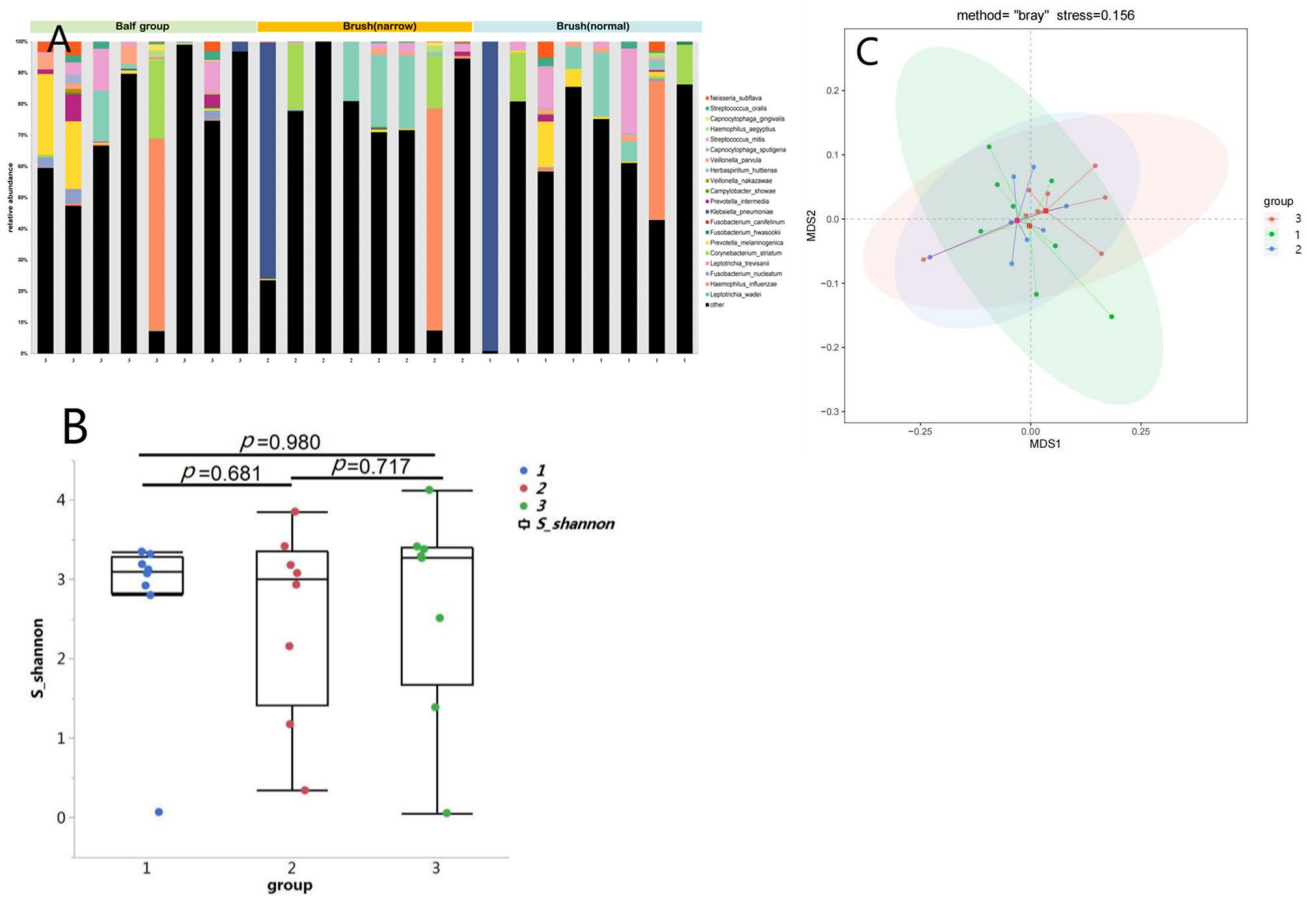
Tracheal microbiome profiling. **A** The stacked bar graph showed the top 20 species s in the scar PSB, scar-free PSB and BLA samples. **B** Comparison of alpha diversity in the microbiome among scar PSB, scar-free PSB and BLA samples. **C** Comparison of beta diversity in the microbiome among scar PSB, scar-free PSB and BLA samples. 1: scar PSB group; 2: scar-free PSB group; 3: BLA group. BAL: broncho-alveolar lavage; PSB: protected specimen brush

A total of 24 samples were collected, including 8 PSB samples from tracheal scar sites, 8 PSB samples from tracheal scar-free sites and 8 BAL samples. The etiologies of endotracheal intubation were varied, including trauma, acute myocardial infarction, severe pneumonia, pesticide poisoning and intracranial tumors. The days of intubation varied from 3 days to 15 days. The time from removing the endotracheal tube to the appearance of tracheal stenosis varied from 2 weeks to 2 months (Table 1). At the species level, the top 20 bacterial species among all samples are represented in Fig. 1A, with the top four consisting of Neisseria subflava, Streptococcus oralis, Capnocytophaga gingivals, and Haemophilus aegyptius.

**Table 1:** clinical data. BAL: broncho-alveolar lavage; PSB: protected specimen brush; Days of intubation: days of endotracheal intubation; Time of extubation: time from pulling out the endotracheal intubation to the occurrence of tracheal stenosis

Patient ID	sample ID	Group	Etiology of endotracheal intubation	Days of intubation	Time of extubation	Age	Gender
1	MDY124-14D0002L-DNA-B44-20210817	broncho-alveolar lavage (BAL)	multiple trauma	10 days	two months	21	male
	MDY124-14D0004Z-DNA-B30-20210817	non-scar PSB					
	MDY124-14D0003Z-DNA-B29-20210817	scar PSB					
2	MDL001-14D2136026Z-DNA-D44-20210831	broncho-alveolar lavage (BAL)	traumatic splenic rupture	7 days	two months	55	male
	MDL001-14D2136025Z-DNA-D43-20210831	non-scar PSB					
	MDL001-14D2136026Z-DNA-D44-20210831	scar PSB					
3	MDY143-14D0022G-DNA-A07-20211229	scar PSB	acute myocardial infarction	3 days	two weeks	67	male
	MDY143-14D0020L-DNA-A05-20211229	broncho-alveolar lavage (BAL)					
	MDY143-14D0021G-DNA-A06-20211229	non-scar PSB					
4	MDY143-14D0003G-DNA-A31-20211015	non-scar PSB	pesticide poisoning	4 days	four weeks	23	female
	MDY143-14D0002G-DNA-A30-20211015	scar PSB					
	MDY143-14D0001G-DNA-A29-20211015	broncho-alveolar lavage (BAL)					
5	MDY143-14D0010G-DNA-D06-20211205	non-scar PSB	severe pneumonia	15 days	two months	75	female
	MDY143-14D0009L-DNA-D23-20211205	broncho-alveolar lavage (BAL)					
	MDY143-14D0011G-DNA-D07-20211205	scar PSB					
6	MDY143-14D0013G-DNA-A31-20211213	scar PSB	intracranial tumors	4 days	two months	21	male
	MDY143-14D0023L-DNA-D09-20220129	broncho-alveolar lavage (BAL)					
	MDY143-14D0014G-DNA-A32-20211213	non-scar PSB					
7	MDL001-14D2136048L-DNA-D25-20210901	broncho-alveolar lavage (BAL)	Head and face trauma	10 days	two weeks	49	male
	MDL001-14D2136047Z-DNA-D24-20210901	non-scar PSB					
	MDL001-14D2136046Z-DNA-D23-20210901	scar PSB					
8	MDY143-17D0003G-DNA-B05-20211231	non-scar PSB	pesticide poisoning	4 days	four weeks	49	female
	MDY143-17D0001L-DNA-B03-20211231	broncho-alveolar lavage (BAL)					
	MDY143-17D0002G-DNA-B04-20211231	scar PSB					

### Diversity comparison among 3 groups

The alpha and beta diversity among tracheal scar PSB and scar-free PSB and BAL samples were compared. There was no significant difference in alpha diversity as measured by the Shannon index among the 3 groups (P = 0.98, Fig. 1B). Similarly, no significant difference in beta diversity was found (P = 0.67, Fig. 1C). Further LEfSe analysis showed no significant differences, indicating no differences in lung microbiota among the 3 groups.

### LC‒MS metabolomics results

**Fig. 2:**
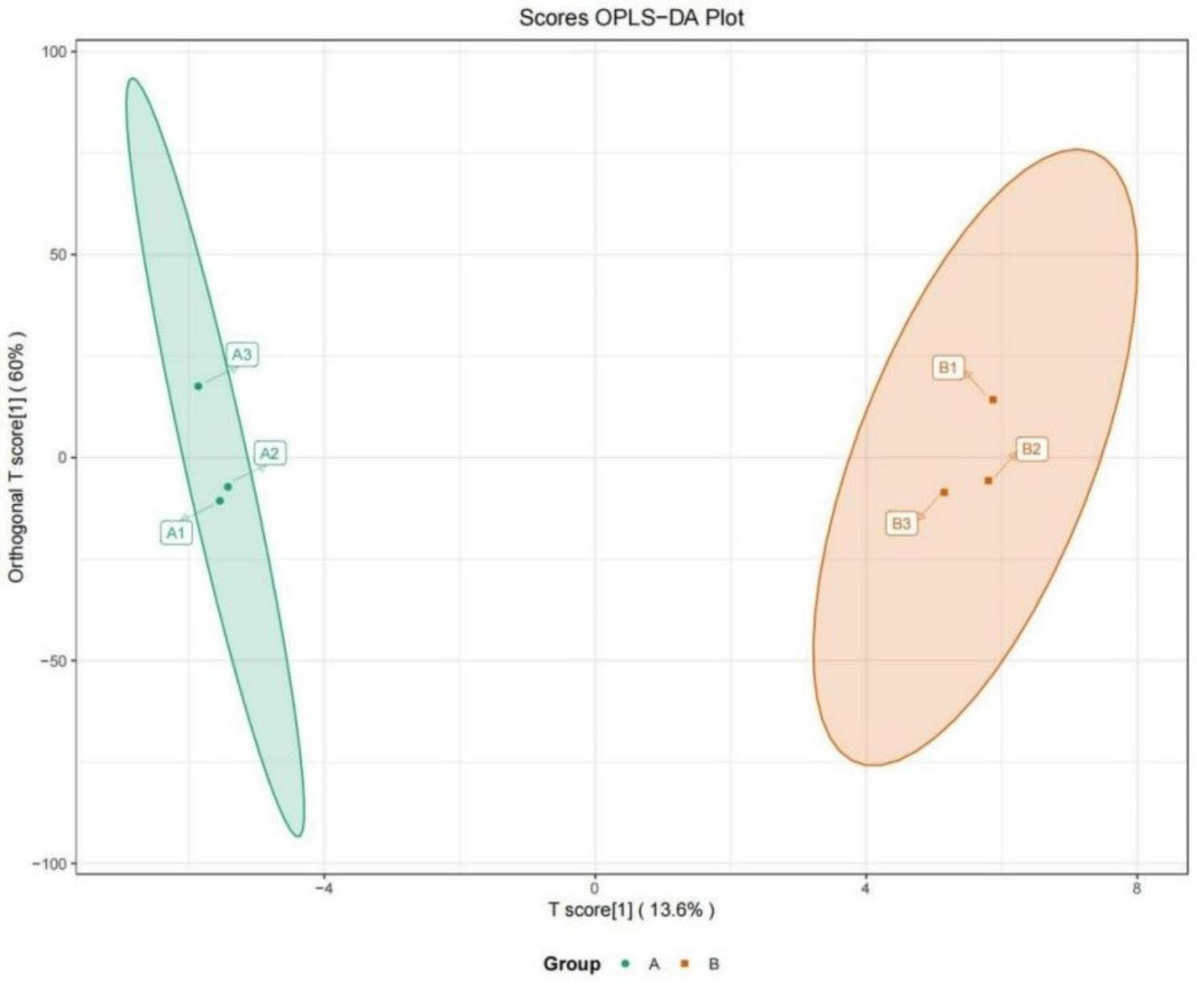
Metabolomic analysis of PSB samples. OPLS-DA model indicates a signifificant difference between scar (A group) and scar-free PSB (B group) (R2Y = 99.8%, Q2Y = 67.7%). BAL: broncho-alveolar lavage; PSB: protected specimen brush

**Fig. 3:**
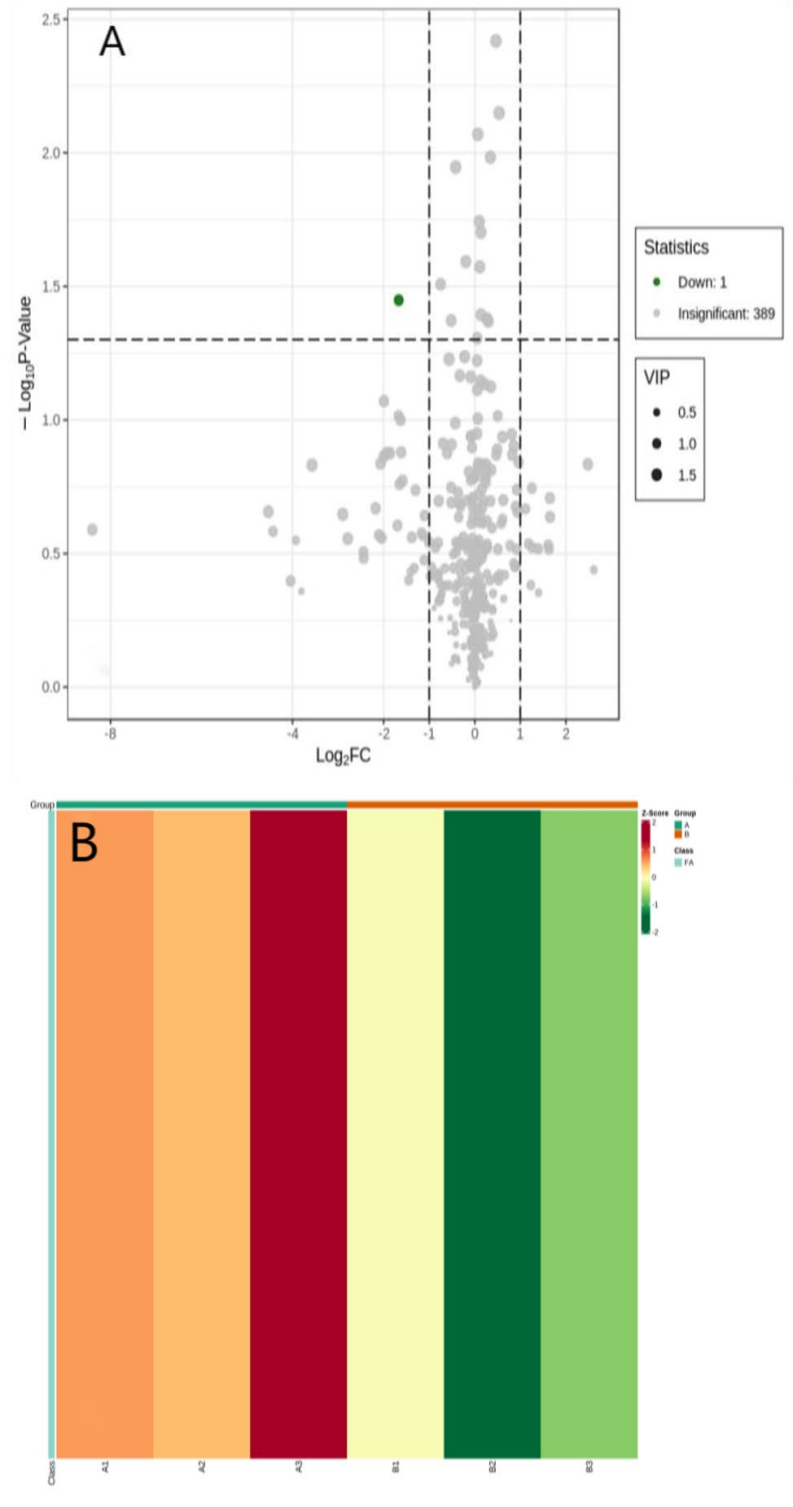
** A** Based on the specified screening criteria, the volcanic map showed a significantly down-regulated metabolite carnitine between the scar and scar-free PSB. **B** Heatmap showed that difference in carnitine content between scar (A group) and scar-free PSB (B group) (a red color indicates a high carnitine level). BAL: broncho-alveolar lavage; PSB: protected specimen brush

**Fig. 4:**
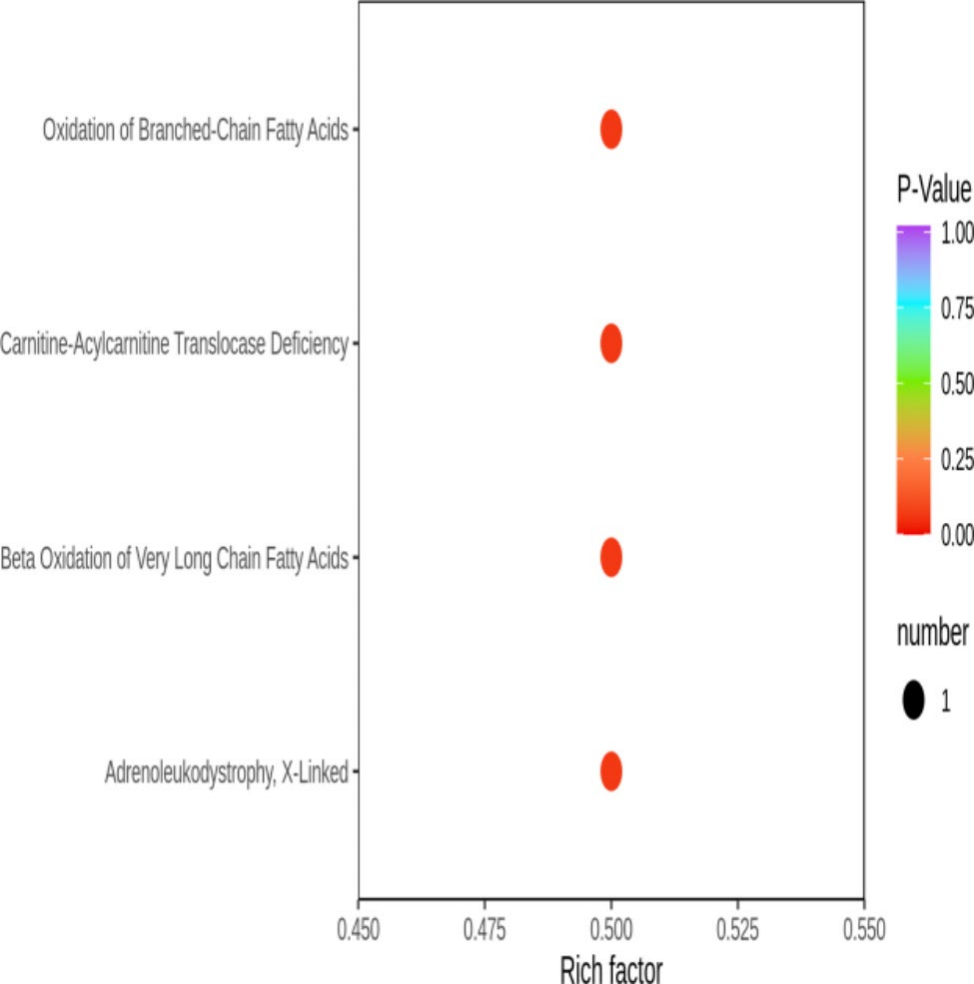
A total of 4 pathways were detected by pathway enrichment analysis of carnitine using HMDB database. Rich factor is the ratio of the number of differentially expressed metabolites in the corresponding pathway to the total number of metabolites annotated by the pathway. The larger the value, the greater the degree of enrichment. The abscissa represents the corresponding Rich factor for each pathway, the ordinate is the pathway name, and the color of the points is the P-Value, with redder indicating more significant enrichment. The size of the dots represents the number of differentially enriched metabolites

Untargeted metabolomics was performed in 6 PSB samples, including 3 scar PSB and 3 scar-free PSB, using high-performance liquid chromatography‒mass spectrometry (LC‒MS). The OPLS-DA plot score showed that scar sites were obviously separated from scar-free sites (Fig. 2). Permutation tests verified that the PLS-DA model is stable and not overfit (R2Y = 99.8%, Q2Y = 67.7%). Based on VIP (variable importance in projection) ≥ 1, fold change ≥ 2 and p value < 0.05, only one statistically significant metabolite, carnitine, was identified (Fig. 3A). We found that carnitine levels in tracheal scar sites were significantly lower than those in scar-free sites (Fig. 3B). Pathway enrichment analysis of carnitine in tracheal scar sites revealed significant enrichment in fatty acid oxidation (Fig. 4).

## Discussion

To the best of our knowledge, this study is the first to profile the tracheal microbiome and metabolites in ITS patients. There was no significant difference in the alpha and beta diversity of the microbial community among the scar PSB, scar-free PSB and BAL groups. However, metabolomic examination showed that the carnitine content of scar and scar-free sites was significantly different. In addition, pathway enrichment analysis of carnitine revealed significant enrichment in fatty acid oxidation.

In the current study, we performed mNGS sequencing on 24 samples from 8 ITS patients. We found that Neisseria subflava, Streptococcus oralis, Capnocytophaga gingivals and Haemophilus aegyptius were the most abundant species. Most of them are common oral species, suggesting that upper respiratory flora can enter the lower respiratory tract by microinhalation. The pulmonary microbiota is determined by the balance between immigration from the upper respiratory tract, local replication and elimination [25]. In patients with tracheal stenosis, structural disturbances of the local trachea may affect the removal and replication of local microbiota and further lead to disordered microbiota. Changes in tracheal microbiota composition may activate the local inflammatory immune response and further aggravate tracheal stenosis [26, 27]. However, it is still unclear whether airway flora disorder triggers inflammation or whether it is a consequence of airway disease. In the present study, we also found that the highest proportion of microbiota isolated from most samples were classified as “other”. Possible explanations were as follows: First, compared with the gastrointestinal tract with the richest microbial community in the human body, the types and abundance of microorganisms in the respiratory tract were relatively few. In addition, mNGS can capture all DNA or RNA sequences in the sample and identify the full spectrum of microorganisms, including bacteria, viruses, fungi and parasites. Therefore, the amount of data generated by each sequencing of mNGS is enormous. We chose to show the 20 species with the highest average relative abundance among all samples, and the relative abundance of other species varied greatly among different samples. Taken together, these factors may cause the microorganisms isolated from most samples to be classified as “other”.

Recent studies emphasized the relationship between microbiota dysbiosis and pulmonary diseases [27, 28]. A study by Gelbard et al. demonstrated that mycobacterial species were associated with idiopathic subglottic stenosis [19]. Furthermore, in the research by Hillel et al., 16 S rRNA amplicon sequencing showed that the tracheal microbiota diversity of scar sites was decreased compared to that of scar-free sites in all etiology groups, which included ITS and idiopathic subglottic stenosis. Unfortunately, this study did not compare microbial diversity between scar and scar-free sites in the ITS subgroup [7]. In our study, we found no significant difference in alpha and beta diversity of microbial community, which was different from the studies by Gelbard et al [19] and Hillel et al [7]. Possible reasons were as follows: First, the subjects included were different. The two studies cited in our article compared idiopathic subglottic tracheal stenosis, ITS and healthy control group. However, we only compared the scar and scar-free sites of ITS. Second, the sequencing techniques utilized were different. The sequencing technology used in our study was mNGS, which identified the full spectrum of microbes, including bacteria, viruses, fungi, and parasites. Its advantage is that microbial identification can be accurate to the species level [29], which enriches our understanding of the respiratory microecology of ITS patients. However, 16 S sequencing mainly identifies and classifies bacteria at the genus level. The species measured by 16 S sequencing were more abundant at the genus level [30]. Therefore, there are some differences between the two analysis objects. Third, there may be no statistical difference in microbial diversity among scar PSB, scar-free PSB and BAL samples, because the spatial variation of microbiota within an individual was significantly smaller than variation across individuals [25].

Metabolomic analysis of PSB was used to screen metabolites associated with tracheal stenosis. Finally, only one statistically significant metabolite, carnitine, was identified. Carnitine, an amino acid derivative, is found in nearly all cells of the body and plays an important role in antioxidant stress and energy metabolism [31]. In recent years, increasing evidence has confirmed that carnitine provides protection against inflammatory reactions and oxidative stress injury [32, 33]. For example, it has been reported that carnitine plays a protective role in inflammatory bowel disease by suppressing immune cell activation, inhibiting oxidative stress, and promoting the integrity of the intestinal epithelium [34]. Moreover, a clinical study showed that supplementing carnitine could improve the condition of chronic obstructive pulmonary disease and acute lung injury [35]. In the present study, we found that carnitine levels in tracheal scar sites were significantly lower than those in scar-free sites, which might suggest the presence of inflammation in the local scar sites.

In addition to the anti-inflammatory and antioxidant effects mentioned above, carnitine also plays an important role in the energy metabolism of cells. Consistently, our enrichment analysis of carnitine revealed significant enrichment in fatty acid oxidation. Metabolic disorders may affect cellular activity, leading to immune disorders and inflammation [35]. Cellular metabolism is very important for the proliferation and quiescent activity of stem cells. Research has found that cells regulate proliferation by switching between different metabolic pathways [36]. Iatrogenic factors such as tracheal intubation may lead to mucosal injury, ischemia and hypoxia, followed by metabolic remodeling of tracheal epithelial cells to support injury and repair [37]. The research by Tsai, H. W et al. revealed that glutamine inhibitors could prevent tracheal scar fibroblast metabolism and proliferation [11]. In addition, fatty acid oxidation in the lung tissue of patients with idiopathic pulmonary fibrosis was abnormal [38]. Moreover, a recent study confirmed that inhibition of fatty acid oxidation impaired the differentiation and repair of airway epithelial cells [39]. In our study, carnitine levels in scar sites were significantly lower than those in scar-free sites, suggesting that fatty acid metabolism in scar sites was blocked, which might explain the aberrant inflammatory activation and abnormal healing response in scar tissue.

Our research had some shortcomings as follows: first, we included a limited sample size; second, due to insufficient remaining sample size, only six PSB samples were subjected to LC‒MS; third, we did not include healthy patients as an external control; fourth, clinical indicators that may be related to tracheal stenosis, such as tracheal intubation type, cuff pressure, intubation days, and antibiotic use during tracheal intubation, were not collected; fifth, we did not include patients with ITS from different hospitals or regions; sixth, because this was a descriptive study, we failed to explore the specific mechanisms of carnitine and fatty acid metabolism in tracheal scar stenosis. Therefore, it is necessary to carry out high-quality prospective researches in the future.

## Conclusion

Our study found that carnitine levels in airway scar tissue were significantly lower than those in nonscar tissue, which might be a new target for the prevention and treatment of iatrogenic airway stenosis in the future.

## Data Availability

All data in this study are provided by zeqin Fan, with contact information: E-mail: fzq687387@163.com. Add: 176 Qingnian Road, Kunming, Yunnan Province, China. Tel: 0871–6515 6650 − 2818

## References

[1] Gelbard A, Francis DO, Sandulache VC (2015). Causes and consequences of adult laryngotracheal stenosis. LARYNGOSCOPE.

[2] Su ZQ, Wei XQ, Zhong CH (2013). [The cause and efficacy of benign tracheal stenosis]. Zhonghua Jie He He Hu Xi Za Zhi.

[3] Zhang J, Wang T, Wang J (2010). Effect of three interventional bronchoscopic methods on tracheal stenosis and the formation of granulation tissues in dogs. Chin MED J-PEKING.

[4] Gnagi SH, Howard BE, Anderson C, Lott DG (2015). Idiopathic subglottic and tracheal stenosis: a Survey of the patient experience. ANN OTO RHINOL LARYN.

[5] Dorris ER, Russell J, Murphy M. Post-intubation subglottic stenosis: aetiology at the cellular and molecular level. EUR RESPIR REV 2021;30. 10.1183/16000617.0218-2020 PMC9489001 33472959

[6] Yin LX, Motz KM, Samad I (2017). Fibroblasts in hypoxic conditions mimic Laryngotracheal Stenosis. OTOLARYNG HEAD NECK.

[7] Hillel AT, Tang SS, Carlos C et al. Laryngotracheal Microbiota in Adult Laryngotracheal stenosis. MSPHERE 2019;4. 10.1128/mSphereDirect.00211-19 PMC6495342 31043518

[8] Shah R, Bunyavanich S (2021). The airway microbiome and pediatric asthma. CURR OPIN PEDIATR.

[9] Natalini JG, Singh S, Segal LN (2023). The dynamic lung microbiome in health and disease. NAT REV MICROBIOL.

[10] Aronson MR, Ali AGS, Gehret PM, Jacobs IN. In: Gottardi R, editor. Drug-eluting endotracheal tubes for preventing bacterial inflammation in Subglottic Stenosis. LARYNGOSCOPE; 2021. 10.1002/lary.29769 34319583

[11] Tsai HW, Motz KM, Ding D (2020). Inhibition of glutaminase to reverse fibrosis in iatrogenic laryngotracheal stenosis. LARYNGOSCOPE.

[12] Nakagishi Y, Morimoto Y, Fujita M (2005). Rabbit model of Airway Stenosis Induced by scraping of the Tracheal Mucosa. Laryngoscope.

[13] Puyo CA, Dahms TE (2012). Innate immunity mediating inflammation secondary to endotracheal intubation. Archives of otolaryngology–head & neck Surgery.

[14] Gelbard A, Katsantonis NG, Mizuta M (2016). Idiopathic subglottic stenosis is associated with activation of the inflammatory IL-17A/IL-23 axis. LARYNGOSCOPE.

[15] Davis RJ, Lina I, Green B (2021). Quantitative Assessment of the Immune Microenvironment in patients with iatrogenic laryngotracheal stenosis. OTOLARYNG HEAD NECK.

[16] Feng Q, Chen W, Wang Y. Gut microbiota: an integral moderator in Health and Disease. FRONT MICROBIOL 2018;9. 10.3389/fmicb.2018.00151 PMC5826318 29515527

[17] Bowerman KL, Rehman SF, Vaughan A (2020). Disease-associated gut microbiome and metabolome changes in patients with chronic obstructive pulmonary disease. NAT COMMUN.

[18] Zhao F, An R, Wang L, Shan J, Wang X (2021). Specific gut microbiome and serum metabolome changes in Lung Cancer Patients. FRONT CELL INFECT MI.

[19] Gelbard A, Katsantonis NG, Mizuta M (2017). Molecular analysis of idiopathic subglottic stenosis for Mycobacterium species. LARYNGOSCOPE.

[20] Myer CR, O’Connor DM, Cotton RT (1994). Proposed grading system for subglottic stenosis based on endotracheal tube sizes. ANN OTO RHINOL LARYN.

[21] Shen H, Shen D, Song H (2021). Clinical assessment of the utility of metagenomic next-generation sequencing in pediatric patients of hematology department. INT J LAB HEMATOL.

[22] Zhang R, Zhuang Y, Xiao Z et al. Diagnosis and surveillance of neonatal infections by Metagenomic Next-Generation sequencing. FRONT MICROBIOL 2022;13. 10.3389/fmicb.2022.855988 PMC8989347 35401464

[23] Jiang H, Wu C, Xu J (2021). Bacterial and fungal infections promote the bone Erosion progression in Acquired Cholesteatoma revealed by Metagenomic Next-Generation sequencing. FRONT MICROBIOL.

[24] Luo W, Hu T, Luo L, Li Y. Pulmonary sequestration with aspergillus infection presenting as massive hemoptysis and hemothorax with highly elevated carcinoembryonic antigen in pleural effusion that mimics advanced lung malignancy. EUR J MED RES 2021;26. 10.1186/s40001-021-00519-5 PMC8146657 34034813

[25] Dickson RP, Erb-Downward JR, Freeman CM (2015). Spatial variation in the Healthy Human Lung Microbiome and the adapted Island Model of Lung Biogeography. ANN AM THORAC SOC.

[26] Segal LN, Clemente JC, Tsay JC (2016). Enrichment of the lung microbiome with oral taxa is associated with lung inflammation of a Th17 phenotype. NAT MICROBIOL.

[27] O’Dwyer DN, Ashley SL, Gurczynski SJ (2019). Lung microbiota contribute to pulmonary inflammation and Disease Progression in Pulmonary Fibrosis. AM J RESP CRIT CARE.

[28] Yang D, Xing Y, Song X, Qian Y (2020). The impact of lung microbiota dysbiosis on inflammation. Immunology.

[29] Chiu CY, Miller SA (2019). Clinical metagenomics. NAT REV GENET.

[30] Ranjan R, Rani A, Metwally A, McGee HS, Perkins DL (2016). Analysis of the microbiome: advantages of whole genome shotgun versus 16S amplicon sequencing. BIOCHEM BIOPH RES CO.

[31] Wang Z, Liu Y, Liu G, Lu H, Mao C (2018). L -Carnitine and heart disease. LIFE SCI.

[32] Bae JE, Kim JB, Jo DS et al. Carnitine protects against MPP(+)-Induced neurotoxicity and inflammation by promoting primary ciliogenesis in SH-SY5Y cells. CELLS-BASEL 2022;11. 10.3390/cells11172722 PMC9454591 36078130

[33] Shaforostova EA, Gureev AP, Volodina DE, Popov VN (2022). Neuroprotective effect of mildronate and L-carnitine on the cognitive parameters of aged mice and mice with LPS-induced inflammation. METAB BRAIN DIS.

[34] Fortin G (2011). l-Carnitine and intestinal inflammation.

[35] Wang M, Wang K, Liao X (2021). Carnitine palmitoyltransferase system: a New Target for anti-inflammatory and anticancer therapy?. FRONT PHARMACOL.

[36] Chandel NS, Jasper H, Ho TT, Passegué E (2016). Metabolic regulation of stem cell function in tissue homeostasis and organismal ageing. NAT CELL BIOL.

[37] Liu G, Summer R (2019). Cellular Metabolism in Lung Health and Disease. ANNU REV PHYSIOL.

[38] Geng J, Liu Y, Dai H, Wang C (2021). Fatty acid metabolism and idiopathic pulmonary fibrosis. FRONT PHYSIOL.

[39] Crotta S, Villa M, Major J (2023). Repair of airway epithelia requires metabolic rewiring towards fatty acid oxidation. NAT COMMUN.

